# Breast Cancer Stem Cells and Tumor Heterogeneity: Characteristics and Therapeutic Strategies

**DOI:** 10.3390/cancers16132481

**Published:** 2024-07-07

**Authors:** Aleksandra Romaniuk-Drapała, Ewa Totoń, Magdalena Taube, Malgorzata Idzik, Błażej Rubiś, Natalia Lisiak

**Affiliations:** Department of Clinical Chemistry and Molecular Diagnostics, Poznan University of Medical Sciences, Collegium Pharmaceuticum, Rokietnicka Str. 3, 60-806 Poznan, Poland; aromaniuk@ump.edu.pl (A.R.-D.); etoton@ump.edu.pl (E.T.); magdalena.taube@ump.edu.pl (M.T.); gosiq254@gmail.com (M.I.); blazejr@ump.edu.pl (B.R.)

**Keywords:** breast cancer subtypes, BCSC, biomarkers, signaling pathways, therapy

## Abstract

**Simple Summary:**

Breast cancer stem cells (BCSCs) are a small population of cells in breast cancer tumors, playing a putative role in the cancer’s progression. BCSC biomarkers are associated with cells’ enhanced growth, adhesion, migration, and invasion potential and are responsible for poor outcomes. Due to the vast heterogeneity of breast cancer signaling pathways and associated therapeutic targets, treatment strategies vary and depend on the molecular subtype of BC. Nevertheless, drugs affecting the Wnt, Notch, Hedgehog, PI3K/Akt/mTOR, and HER2 signaling pathways have been effectively applied in BCSC elimination strategies. The complexity of the characteristics of each molecular subtype of breast cancer, with an emphasis on the involvement of breast cancer stem cells in the development, metastasis, and recurrence of the tumor, requires greater understanding. Continuous updating of knowledge and the development of new therapies will allow the effective elimination of cancer cells and the rapid recovery of patients.

**Abstract:**

Breast cancer is one of the most frequently detected malignancies worldwide. It is responsible for more than 15% of all death cases caused by cancer in women. Breast cancer is a heterogeneous disease representing various histological types, molecular characteristics, and clinical profiles. However, all breast cancers are organized in a hierarchy of heterogeneous cell populations, with a small proportion of cancer stem cells (breast cancer stem cells (BCSCs)) playing a putative role in cancer progression, and they are responsible for therapeutic failure. In different molecular subtypes of breast cancer, they present different characteristics, with specific marker profiles, prognoses, and treatments. Recent efforts have focused on tackling the Wnt, Notch, Hedgehog, PI3K/Akt/mTOR, and HER2 signaling pathways. Developing diagnostics and therapeutic strategies enables more efficient elimination of the tumor mass together with the stem cell population. Thus, the knowledge about appropriate therapeutic methods targeting both “normal” breast cancer cells and breast cancer stem cell subpopulations is crucial for success in cancer elimination.

## 1. Introduction

Breast cancer (BC) became the most common cancer globally as of 2022, accounting for 11.6% of all new annual cancer cases worldwide in females and 6.9% of deaths, according to the Global Cancer Observatory [1]. Most breast cancer cases are associated with older age, which is the most important risk factor. Other key factors include early menarche, late age of the first childbirth, late menopause age, long-term hormonal contraception and hormone replacement therapy, unhealthy diet, low daily physical activity, overweight and obesity, exposure to ionizing radiation, some benign proliferative diseases of the breast, and specific gene mutations (mainly *BRCA1*, *BRCA2*, or *TP53*), with family history of breast cancer (especially in young patients) [2,3]. The most important prognostic factors in BC involve tumor size, histological type, grade of cancer, identification of metastases in the axillary lymph nodes, and the number of nodes affected by metastases. In addition, infiltration of peritumoral lymphatic and venous vessels, the presence of estrogen (ER), progesterone (PR), and epidermal growth factor receptor 2 (HER2), the Ki67 proliferation index, and a panel of biomarkers that determine the molecular subtype of breast cancer are also assessed [4,5]. This high tumor heterogeneity is attributed to differences in cancer cells’ genomic, epigenomic, transcriptomic, and proteomic characteristics. These factors affect tumor properties such as proliferation, apoptosis, metastasis, and therapeutic response [6]. One common factor contributing to the aggressiveness, progression, and poor response to treatment is breast cancer stem cells (BCSCs) [7]. BCSCs represent a separate subpopulation of tumor cells characterized by the ability to differentiate into non-BCSCs and a remarkable capacity for self-renewal. BCSCs can be distinguished by the low expression of differentiation markers and the high expression of CSC markers. Moreover, they demonstrate resistance to conventional therapy due to specific signaling pathway activity and high tumorigenesis potential [8]. Drug resistance is a major challenge in breast cancer treatment. Accumulating studies indicate that CSCs are responsible for treatment resistance and cancer metastasis, causing relapses. Self-renewal ability is tightly regulated and plays a critical role in preventing the differentiation of CSCs [9]. Most promising therapeutic strategies are based on targeting and maintaining stemness signaling pathways due to the crucial role of BCSCs in driving breast cancer aggressiveness. Additionally, BCSC elimination could improve drug efficacy and reverse drug resistance to benefit BC patients. However, the diversity of biological and genetic features within the breast cancer cell population adds to a significant diagnostic and therapeutic challenge.

Mastering the systematization of breast cancer subtypes and the specific BCSC biomarkers associated with them will help create an effective treatment strategy for all breast cancer variants. In this review, we will discuss BC characteristics and classification and the main signaling pathways of BCSC, which could be a target of therapy.

## 2. Breast Cancer Characteristics and Classification

According to the European Society of Medical Oncology (ESMO) recommendation, patients with newly diagnosed or recurrent metastatic breast cancer (MBC) are diagnosed based on a biopsy for histology confirmation and also the status of ER, PR, and HER2 receptors. Other biomarkers that are assessed include the status of germline BRCA1/2 mutation in HER2-negative MBC, the status of programmed death-ligand 1 (PD-L1) in TNBC, and also PIK3CA status in ER/PR-positive, HER2-negative MBC [10].

However, the classification of breast cancer has evolved over the years. The widely accepted and most common classification is based on assessing the expression of the hormone receptors, e.g., estrogen (ER), progesterone (PR), and human epidermal growth factor (HER2) by immunohistochemical methods. This classification scheme divides breast cancer into four molecular subtypes: luminal A, luminal B, HER2-positive, and basal-like (with triple-negative breast cancer) [11].

Normal breast cells express ER, PR [12], and HER2, stimulating cell growth [13]. However, not all breast cancer cells show hormone receptor (HR) expression (ER and PR, ca. 66%), while only 20 to 30% of breast cancer cases show elevated HER2 levels [14]. Luminal A breast cancer shows estrogen and progesterone receptor expression, does not express HER2, and is characterized by a low level of the proliferation marker protein Ki-67 [15]. Ki-67 reflects the extent of the proliferative activity of tumor cells and is a reliable marker of more aggressive breast cancers. This subtype of breast cancer is characterized by the lowest grade of malignancy and the best prognosis (over 80% 5-year survival) that is associated with anticipated better response to therapy [16,17,18].

Luminal B breast cancer, similar to the luminal A subtype, is estrogen and progesterone-dependent, with HER2 positive or negative, while showing high levels of Ki-67 protein. This cancer subtype has a slightly higher proliferation potential and slightly worse prognosis compared to the luminal A subtype (over 70% 5-year survival) [19].

The HER2-positive subtype of breast cancer shows an expression of the HER2 receptor, while there is no expression of ER and PR receptors. This molecular subtype of breast cancer, apart from being characterized by faster growth, also has a worse prognosis: approximately 50–60% 5-year survival [20,21].

Basal breast cancer (also called basal-like breast cancer (BLBC)) lacks the expression of ER, PR, and HER2. This subtype of cancer is more common in BRCA1 mutation carriers as well as in younger individuals and black women [22]. BLBC cells are similar to triple-negative breast cancer (TNBC) cells but show alterations in the profile of proteins that triple-negative breast cancers usually do not demonstrate. BLBC is positive for the basal markers such as cytokeratin (CK) proteins of cytoskeletal intermediate filaments, responsible for withstanding mechanical stress CK5/6 and/or CK17 and/or CK14 [23,24]. It is worth noting that not all triple-negative BCs are basal-like, and vice versa; they are very similar but distinct breast cancer subtypes. Triple-negative BC is more common in women under 40 and of Asian or African descent. However, BLBC is more common in younger women of African origin [25,26].

Among various subtypes of breast cancer, the triple-negative breast cancer subtype (TNBC) is the most diverse. There are a few classifications of TNBC, the first proposed by Lehmann in 2011, the next presented in 2015 by Burstein, and the FUSCC (the Fudan University Shanghai Cancer Center) classification from 2016. Lehmann divided TNBC into six subtypes based on different gene expression profiles, response to standard treatments, and prognosis: basal-like 1 (BL1), basal-like 2 (BL2), immunomodulatory (IM), mesenchymal (M), mesenchymal stem-like (MSL), luminal androgen receptor subtype (LAR), and mesenchymal–epithelial transition subtype (MET) [27,28,29,30]. Four years later, based on gene expression profiles, Burstein et al. reduced this classification and divided TNBC into four subtypes: basal-like immune-activated (BLIA), basal-like immune-suppressed (BLIS), mesenchymal-like subtype (MES), and luminal androgen receptor subtype. Moreover, the FUSCC classification of TNBC distinguishes the following four types: IM, LAR, MES, and BLIS, according to integrating transcriptome profiles of mRNA and lncRNA [31]. Due to the diversity within this group, each subtype has different characteristics, prognoses, and treatment effectiveness. Therefore, TNBC subtyping is of value for prioritizing patients for personalized medicine. However, we still lack a laboratory tool to enable this classification in the routine protocol [32].

Characteristics of individual breast cancer subtypes, including frequency, immunohistochemistry characteristics, and prognosis according to global statistics, are presented in Table 1.

## 3. Breast Cancer Stem Cell Subpopulation

Cancer stem cells (CSCs) are a subpopulation of cancer cells with characteristics of normal stem cells. Thus, they can self-renew, differentiate, and possess a selective tumorigenic capacity [34]. Moreover, the presence of CSCs is associated with resistance to chemotherapy and radiotherapy [35]. The origin of CSCs is connected with two models. One of them assumes that normal, long-lived stem cells become malignant through the accumulation of genetic alterations. However, the other model suggests that mutations equip lineage-committed cells with stem characteristics [36]. CSCs, also called tumor-initiating cells (TICs), consist of a small subpopulation of undifferentiated cells constituting only 0.1–1% of a whole cancer mass [37,38].

Breast cancer stem cells (BCSCs), although they constitute a minor subpopulation of breast cancer cells, are responsible for cancer progression [39]. Their first discovery dates back to 2003, when Al-Hajj et al. used the surface markers CD24 and CD44 for identification. The research group concluded that a small percentage of these cells could produce tumors with similar heterogeneity to the original tumor in an immunodeficient mouse model. Moreover, another type of tumor cell (even in higher populations of 10^5^ to 10^6^ cells) could not initiate tumorigenesis in the same kind of mice [40]. In 2007, Ginestier and colleagues identified aldehyde dehydrogenase 1 (ALDH1) as a marker of both normal and tumor mammary stem cells and an indicator of poor prognosis [41].

These biomarkers are associated with enhanced growth, adhesion, migration, and invasion potential of BCSCs, which contribute to poor outcomes [42].

In the following years, CD133, ABCG2, SSEA-3, Nectin-4, MUC1, Lrg5, and CD70 were identified as other biomarkers of BCSCs in breast cancer cell lines [43]. Also, microRNAs were indicated as markers of BCSC subpopulations, regulating signaling pathways responsible for the development and preservation of BCSC (tumor-suppressing: Let-7, miR34, miR200 family, miR30, and miR600, and oncogenic: miR-22, miR155, miR181, and miR221/222 cluster) [44].

### 3.1. Methods for BCSC Detection

However, no single method is universally adopted as the standard for determining the presence of BCSCs due to their complexity and heterogeneity. A routine clinical approach should involve methods that balance sensitivity, specificity, and practicality. Thus, several methods are commonly used, often in combination, to achieve reliable results, and each has its advantages and limitations. The first aspect is a sample type, blood plasma, or biopsy material. Circulating tumor cells (CTCs) are detected in plasma. This is a minimally invasive approach and can be performed repeatedly to monitor disease progression and treatment response. Unfortunately, BCSCs occur in very low concentrations, requiring highly sensitive detection methods. Moreover, directed samples from the biopsy allow a detailed analysis of the tumor microenvironment and cellular composition. However, it is limited by the heterogeneity of the tumor, may not capture the whole spectrum of cancer cells present, and involves invasive procedures [45]. Tumor biopsy samples are used to detect common markers, including CD44, CD24, and ALDH1, by the immunohistochemistry (IHC) method. It is a direct visualization of BCSCs within the tumor context. Nonetheless, it is semi-quantitative and can be subject to interpretation variability. On the other hand, flow cytometry is used based on the expression of these markers to quantify and sort BCSCs. It can be combined with the aldehyde dehydrogenase activity assessment, an ALDEFLUOR assay. This flow cytometry approach requires fresh and viable single-cell suspensions from tumor tissue. Molecular biology techniques (PCR/qPCR, NGS) are used for the quantitative analysis of the expression of BCSC-related genes (e.g., EpCAM, NANOG, SOX2, and OCT4). These are highly sensitive and specific methods used for CTC identification and provide comprehensive profiling of genetic and epigenetic changes, allowing for the identification of rare cell populations. The RNA or DNA can be extracted from tumor samples or circulating tumor cells [46].

The choice of method depends on the clinical or research context, the available resources, and the specific goals of the analysis. A combination of immunohistochemistry and molecular biology techniques is often the most effective approach for a comprehensive assessment of BCSCs. Advances in technology and the development of standardized protocols may facilitate the routine clinical detection of BCSCs in the future.

The expression level of the mentioned biomarkers is different in individual molecular subtypes of breast cancer. Thus, we will discuss these differences in detail in the following caption.

### 3.2. Characteristics of BCSCs in Different BC Molecular Subtypes

CSCs are identified by several biomarkers. There is no consensus on identification patterns or universal markers for CSCs in all types of cancer, although some molecules are frequently shared across entities. The epithelial–mesenchymal transition (EMT) is a process that occurs during regular embryonic development and tissue regeneration. However, alterations of EMT result in malignant properties during tumor development, increase tumor stemness, and are responsible for therapy failure [44,47]. EMT is crucial in the context of BCSCs, which exist in different mesenchymal-like (epithelial–mesenchymal transition [EMT])) and epithelial-like (mesenchymal–epithelial transition [MET])) conditions. Mesenchymal-like BCSCs with CD44+/CD24− characteristics are primarily quiescent and placed at the tumor-invasive front, while epithelial-like BCSCs express ALDH1, are proliferative, and are more centrally located. The gene expression signatures of mesenchymal-like and epithelial-like BCSCs are similar in all breast cancer molecular subtypes and basal and luminal stem cells present in the normal breast. The plasticity of BCSCs enables the transition between EMT- and MET-like states and allows these cells to invade tissues, spread, and grow at sites of metastasis [47]. BCSCs share multiple changes in gene expression involved in the invasion-metastatic cascade, precisely the epithelial-mesenchymal transition pathway. Observed changes include the downregulation of E-cadherin or upregulation of N-cadherin, vimentin, fibronectin, and EMT inducers like Twist, Snail, and Slug. These features are also characteristic of basal and TNBC subtypes of BC [48].

BCSCs exhibit specific patterns of markers connected with CD44, CD24, and ALDH expression levels. The proportions between BCSCs presenting CD44+/CD24− or ALDH1+ are different in the four molecular subtypes of BC. The published data demonstrate that the CD44+/CD24− subpopulation is higher in luminal A BC. In addition, the expression of ALDH1+ is higher in the rest of the subtypes, with a significant overexpression in HER2-positive and most TNBC subtypes. This expression pattern is associated with the ability to form the mammosphere (MS). The ALDH1+ BCSCs form significantly more MS than CD44+/CD24− BCSCs, showing better tumorigenic abilities [49]. Moreover, ALDH1 overexpression is associated with a higher rate of metastasis and recurrence, thus a worse prognosis. However, BCSCs with CD44+/CD24− overexpression are not significantly correlated with metastasis or recurrence [50]. In addition, there is evidence of a direct correlation between NFκB activation and CD44 expression level, resulting in radioresistance and a poor prognosis for patients [51]. Studies based on cell lines and clinical cases may demonstrate different outcomes. Moreover, in clinical cases, it is connected with the stage of breast cancer and the type of studied material. The majority of the studies reveal that the subpopulation of BCSCs is highly presented in the triple-negative breast cancer subtype and basal subtype of breast cancer. These histopathological characteristics have been observed in pre-chemotherapy tissues of breast cancer patients and correlate with higher histological grade, estrogen receptor negativity, high Ki-67 proliferation index, and aggressiveness of the tumor. BCSC presence in patient tissue is mainly associated with tumor recurrence, radiation resistance, and metastasis. Additionally, the pattern of CD44+/CD24− BCSCs is a prognostic marker for metastatic BC [52]. Among the TNBC subtype, there are subtypes, like BL1 and M, which are significantly more enriched in epithelial-like BCS cells. Moreover, differentiated tumor cells were significantly more common not only in BL1 TNBC but also in ER+ and luminal A, while enriched mesenchymal-like BCSC were predominantly found in the ER-, BL2, and M TNBC subtypes. Notably, more highly purified ALDH+CD24−CD44+ BCSCs were found in ER breast cancers [53].

More interesting data indicate the role of high immunoexpression of ALDH1 in early invasive BC. This profile is connected with poor prognostic hallmarks, like a high grade, poor Nottingham Prognostic Index (NPI), and lymph node metastasis. These features are observed in highly proliferative ER+ luminal BC and TNBC subtypes. Moreover, ALDH1 expression was positively correlated with the expression of CD44, TWIST, SOX9, EPCAM, and CD133 at the protein level. This was associated with poor prognostic characteristics and poor outcomes, particularly in the luminal B and TNBC subtypes [54].

Selected miRNA expression level is also a known characteristic of BCSCs. Data demonstrate that miR-200c-141, miR-200b-200a-429, miR-183-96-182, and Let-7 miRNAs are downregulated in human BCSCs. The expression of Let-7 miRNAs decreases in BCSCs and increases with the differentiation of cells. A study on breast cancer cell lines has shown that miR-200c is overexpressed in MCF7 cells, representing the luminal A BC subtype. It reduces transcription factor 8 expression and increases E-cadherin expression, which is associated with tumor dedifferentiation and increased metastatic potential in human carcinomas [55,56]. Additionally, the miR-200 family, miR-9, and miR-155 have been reported to correlate with EMT and BCSC phenotypes. miR-9 is highly expressed in HER2-positive and TNBC subtypes. Moreover, miR-9 is higher in cells with CD44+/CD24− phenotype, vimentin expression, and E-cadherin loss. It is also a prognostic factor for poor disease-free survival. It is documented that overexpression of miR-9 is correlated with poor overall survival (OS) and DFS after 8 years of follow-up [57]. The study reports that the highest expression of miR-155 demonstrates the TNBC subtype and the levels of miR-200a and miR-141 are the highest in the luminal A subtype [58].

The list of characteristic biomarkers of BCSC patterns in different molecular subtypes of BC is presented in Table 2.

## 4. Therapeutic Strategies for the Treatment of Specific Types of Breast Cancer

Due to the extensive heterogeneity of breast cancer, biomedical treatment strategies face great challenges. Currently, the major treatment strategies include surgery, chemotherapy, radiotherapy, immunotherapy, and hormonal therapy. Some may not be effective in completely eradicating the tumor cells and may demonstrate adverse side effects [59]. The choice of treatment depends on the biological features of the cancer, determined by the specific biomarkers attributed to the particular subtype.

Luminal A breast cancer treatment usually includes endocrine therapy with the representative drug tamoxifen. Other drugs used are aromatase inhibitors (AIs, anastrozole, and letrozole) and fulvestrant, in combination with agents such as cyclin-dependent kinase (CDK) inhibitors. Patients with luminal B breast cancer are likely to benefit from chemotherapy (anthracyclines and taxoids), as well as hormone therapy (tamoxifen) and HER2-targeted therapy (trastuzumab) [33]. Treatment options for HER2-positive breast cancer are associated with the stage of cancer and include different methods, like surgery, radiotherapy, chemotherapy, and/or administration of targeted therapy, including trastuzumab, fam-trastuzumab-deruxtecan, pertuzumab, lapatinib, neratinib, and T-DM1 or ado-trastuzumab emtansine [60]. Women with triple-negative breast cancer and most of those with basal-like phenotypes cannot benefit from endocrine therapy or trastuzumab [61,62]. The treatment differs and depends on the TNBC subtype. Thus, this group of cancers can be treated with chemotherapeutic agents (anthracyclines, taxoids, alkylating agents), radiotherapy, and non-HER2-targeted therapy [30,63]. The currently used drugs include cisplatin (DNA damaging agent), dactolisib (imidazoquinoline derivative acting as a PI3K/mTOR inhibitor), bicalutamide (AR inhibitor), and olaparib/niraparib (PARP inhibitors, approved for deleterious germline BRCA-mutated, HER2-negative BC) [64,65,66,67].

### 4.1. BCSC Treatment Strategy

Due to the great diversity of breast cancer signaling pathways and the associated different therapeutic targets, treatment strategies are varied and depend on the molecular subtype of BC. Because chemotherapy and radiotherapy only target the proliferating fraction of tumor cells, BCSCs may avoid systemic therapies, which in turn causes the development of drug resistance. Thus, drug resistance provides BCSCs with a selective advantage over non-CSCs, supporting the “survival of the fittest” hypothesis.

The major signaling pathways regulating BCSC include Wnt, Notch, Hedgehog, PI3K/Akt/mTOR, and HER2. Thus, BCSC therapies are mostly based on blocking these signals [68].

#### 4.1.1. Wnt

In recent decades, an increasing number of studies have demonstrated that Wnt signaling involves, among others, the proliferation, metastasis, shaping of the phenotype, stemness maintenance, and therapeutic resistance of breast cancer [69]. Drug resistance and breast cancer phenotype shaping are mediated by crosstalk between canonical and noncanonical pathways. Canonical Wnt signaling is a β-catenin-dependent pathway mediated by a family of factors associated with breast cancer cell proliferation and stemness maintenance, the T-cell factor (TCF)/lymphatic enhancer factor (LEF) [70]. The Wnt planar cell polarity (PCP) and Wnt–Ca2+ signaling are β-catenin-independent, non-canonical pathways correlated with metastasis [68]. β-Catenin via E-cadherin is responsible for cell–cell adhesion. Numerous studies have shown that these constitutive components of Wnt signaling are modified in breast cancer cells, and this pathway’s activation plays a key role in the development of this type of cancer [70]. The TNBC subtypes, especially M, MSL, and BL-2, are associated with the activation of Wnt signaling, which can be linked to the metaplastic potential of these subtypes [69].

The human IgG2 monoclonal antibody Vantictumab inhibits Wnt signaling by binding to Frizzled receptors (FZD1/2/5/7/8), and it is used in HER2-negative breast cancer treatment [71]. LGK-974 is another canonical Wnt pathway inhibitor, disrupting carboplatin resistance in TNBC, inhibiting cell proliferation, migration, and invasion, inducing apoptosis, and blocking the cell cycle [72,73]. LGK-974 decreases LRP6 phosphorylation in the Wnt-dependent pathway, and the expression of AXIN2, which is the Wnt target gene [74]. Foxy-5 is a drug-mimicking peptide of WNT5A, which triggers cytosolic free calcium signaling without affecting β-catenin activation. It decreases migration and invasion through the mechanism involving DDR1 and alters NFAT [75]. Foxy-5 is used in metastatic ER-negative BC therapy and impairs the migration and invasion of epithelial cancer cells [76]. Cirmtuzumab is a drug used in HER2-negative BC treatment strategy, targeting the receptor tyrosine kinase-like orphan receptor 1 (ROR1), which is a type 1 tyrosine kinase receptor connected with the progression of breast cancer [77,78] (Table 3).

#### 4.1.2. Notch

Notch signaling is responsible for the development and homeostasis of different tissues and organs, and its alterations result in various diseases, including cancer. In malignancies, it can act as a double-edged sword; on the one hand, it can initiate, on the other, restrain cancer progression [79]. Notch 1–3 receptors are overexpressed in luminal cells, while Notch Numb and Numb-like pathway inhibitors are expressed in myoepithelial cells [80]. Notch signaling controls the balance of the luminal and myoepithelial lineages. Lineage tracing studies have shown that Notch signaling drives mammary gland stem cells. Notch signaling was disproportionately activated with increased Notch intracellular domain (NICD) accumulation in multiple breast cancer cell lines and primary samples [81]. Abnormal signaling has also been linked to a subtype of triple-negative breast cancer. Its aggressive, metastatic, and therapy-resistant phenotype is correlated with Notch receptor overexpression. In particular, Notch 4 mutation and overexpression are associated with metastasis and poor prognosis of TNBC, suggesting high activity in BCSCs and involvement in chemotherapy resistance [82]. In particular, Notch–Wnt crosstalk is associated with breast cancer initiation. Conversely, inhibition of Notch signaling has consistently been shown to reduce or abolish the development and/or progression of breast cancer [83]. The protein Nodal, a member of the TGF-beta family, is the regulator of cell fate during tumorigenesis. It is associated with stem cell maintenance, differentiation, and progression of cancer. Nodal staining correlates with breast cancer progression and was shown to be expressed in human breast cancer cell lines, with poor expression in normal mammary epithelial cells [84]. Notch and Nodal signaling have been linked with the presence of aggressive breast cancer types, the BCSCs phenotype, and ABCB1-induced drug resistance [85].

The treatment strategy for BCSCs affecting the Notch pathway comprises the use of γ-secretase inhibitors MK-0752 and RO-4929097. The mechanism involves IL6 blocking Notch–Hey2 signaling and has been used in ER-positive and TNBC breast cancers. Moreover, RO-4929097 decreases the expression of the Notch target genes Hes1, Hey1, and HeyL, inhibiting the growth of cancer cells. Additionally, it blocks the T-cell synthesis of TNF-alpha and increases cancer cells’ radiosensitization [86]. Moreover, treatments with small-molecule γ-secretase inhibitors PF-03084014 (nirogacestat) and RG-4733 have been used in TNBC therapy [85,86,87,88,89,90,91,92]. Other Notch inhibitors used in advanced and metastatic BC are CB-103/LIMANTRAFIN and crenigacest [93,94]. CB-103 is a small molecule inhibitor of protein–protein interaction (PPI), targeting the nuclear Notch transcription complex, which downregulates its target genes (c-MYC, CCND1, HES1) [95]. Crenigacest (LY3039478) is a small inhibitor of NICD release by blocking the activity of the γ-secretase complex [96] (Table 3).

#### 4.1.3. Hedgehog

The Hedgehog (Hh) pathway regulates tissue homeostasis and regeneration but can also be involved in tumorigenesis [97]. Incorrect Hh signaling may lead to different developmental disorders, such as congenital disorders. In adult mammals, its activity decreases but is initiated in the context of tissue repair and tumor growth. Increasing evidence indicates that aberrant activation of this pathway is associated with many aspects of tumorigenesis, including tumor initiation, progression, drug resistance, and metastasis [98].

Hh signaling has been linked to breast cancer, especially HR-positive and TNBC subtypes [99]. In human BCSCs, Hh pathway initiation activates glioma-associated oncogene (GLI) transcription factors, and its overexpression is associated with worse outcomes in BC patients. Noncanonical GLI1 activation is involved in estrogen-driven promotion of breast cancer stem cell proliferation and epithelial-mesenchymal transition [100]. As in other cancers, in hormone-resistant cell lines, Hh signaling is activated through the PI3K/AKT pathway and is responsible for chemotherapy resistance in TNBC by different mechanisms, including selective proliferation of BCSCs [101]. Moreover, activation of both Hh and Wnt pathways is a poor prognostic marker in the TNBC subtype [99]. The treatment strategy linked to the Hedgehog signaling pathway comprises drugs like Vismodegib (in HR+ and TNBC) and Sonidegib (in ER-, HER2-negative, TNBC). These orally active small molecule (SMO) inhibitors eliminate BCSCs by blocking Hh signaling through inhibition of the Smoothened, leading to GLI inactivation [102,103]. Moreover, Sonidegib is a drug–drug interaction and multidrug resistance (ABCB1 and ABCG2) modulator, enhancing the cytotoxicity of applied drugs [104]. Another drug, GANT61, inhibits Hh signaling by the GLI1 and GLI2 transcription factors and is an effector of the Hh pathway used in ER-positive and TNBC breast cancers [105,106,107,108]. GANT61 impairs stem cell phenotypes and sphere-forming capacity and decreases breast cancer migration and invasion [109]. Moreover, GANT61 in conjunction with Vismodegib decreased the tumor growth, and GANT61 in combination with paclitaxel inhibited CSC growth and activity [99,110,111] (Table 3).

#### 4.1.4. PI3K/Akt/mTOR

The phosphatidylinositol 3-kinase (PI3K)/protein kinase B (AKT)/mammalian target of rapamycin (mTOR) signaling pathway is one of the most significant pathways in cancer stem cells. It is responsible for the proliferation, differentiation, EMT process, migration, and maintenance of stemness of cancer cells. Therefore, targeting the PI3K/Akt/mTOR signaling might be an effective strategy for cancer elimination. Mutations in the PI3K/Akt/mTOR pathway are frequent in BC (20–40%, especially HR+) and correlate with aggressive tumor behavior and endocrine and anti-HER2-targeted therapy resistance [112,113].

Therapy targeting this pathway uses mTOR inhibitor Everolimus, which strikes crucial cancer cell proteins responsible for proliferation, adhesion, and invasion, and is used in accordance with ESMO treatment recommendations for treatment strategies in HR+, HER2-negative BC in combination with fulvestrant or exemestane [114,115,116,117,118,119]. It binds to the mTOR intracellular receptor, FKBP12, and inhibits its downstream pathways [120]. The GDC-0941 (pictilisib), an orally bioactive inhibitor of I class PI3K isoforms, is used in ER-negative and HER2-negative BC [121,122] and interestingly, in combination with docetaxel, reveals anticancer activity in HER2+ BC models [123]. In addition, alpelisib (BYL719), an α-specific PI3K inhibitor, strongly and selectively inhibits p110α in PIK3CA-altered luminal BC [124,124,125,126]. The combination with fulvestrant has been approved by the FDA for the treatment of HR+ and HER2+ metastatic breast cancer [127]. Another group of PI3K/Akt pathway inhibitors are XL147, NVP-BKM120, LY-294002, and Perifosine, and they have been used in the treatment of HR-positive and HER2-negative cases [128,129]. XL147 (SAR245408) is a highly selective inhibitor of I class PI3Ks, blocks the formation of PIP3 in the membrane, and inhibits phosphorylation of AKT, S6, and p70S6K [130]. LY-294002 increases the formation of the nuclear foci of γ-H2AX and downregulates BRCA1 and RAD51 [131]. Perifosine is also an AKT inhibitor, decreasing UCHL3 deubiquitination activity and inhibiting HR-mediated DSB repair by increasing RAD51 ubiquitination and blocking the function of Rad51 [132].

Another example is flubendazole, Akt, and STAT inhibitors, regulating autophagy and mitophagy, applied in TNBC treatment [133,133,134]. Flubendazole decreases the cancer stem cell subpopulation, formation of mammospheres, expression of stemness genes (*c-MYC*, *OCT4*, *SOX2*, *NANOG*), and expression of EMT markers (N-cadherin, vimentin). Additionally, it induces cell cycle arrest in the G2/M phase. Moreover, it effectively enhances the cytotoxicity of drugs used in breast cancer treatment, e.g., doxorubicin and fluorouracil [135] (Table 3).

#### 4.1.5. HER2

**Figure 1:**
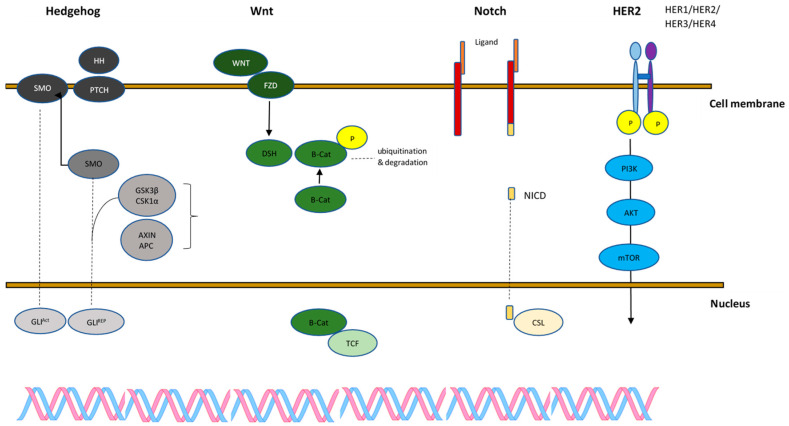
Pathways associated with the signaling of BCSCs. Canonical activation of Hedgehog (Hh) signaling is initiated by the binding of the Hh ligand to PTCH1, which exits the primary cilium (PC), relieving SMO’s inhibition and resulting in SMO’s translocation into the PC. In the lack of HH ligands, PTCH1 inhibits SMO by disturbing its entry into the PC. GLI2 and GLI3 are sequestered in the cytoplasm by SUFU and phosphorylated by PKA, CK1, and GSK3β. GLI1 is fully degraded, while GLI3 and GLI2 undergo fragmentary proteasome degradation. This leads to the creation of GLI3/2R, which moves into the nucleus, resulting in the inhibition of the GLI target gene transcription. GLI2 and GLI3 processing is disturbed by active SMO, which initiates their dissociation from SUFU, and translocation of full-length, active GLI (GLIACT) into the nucleus, resulting in the activation of GLI target gene transcription. Cadherins modulate Wnt/β-catenin signaling. The level of free β-catenin in the cytoplasm directly affects the degree of β-catenin accumulation in the nucleus. Free β-catenin has a short half-life due to its phosphorylation by GSK3 and destruction upon binding to the adenomatous polyposis coli (APC) and Axin proteins. GSK3 activity is reduced by disheveled (DSH) when the Wnt growth factor binds to its surface receptor Frizzled (FZD). Notch signaling DSL (Delta/Serrate/Lag-1) ligands binding changes the conformation of the Notch receptor. The ADAM family arbitrates the first cleavage, which leads to the separation of the extracellular domain. A second cleavage then occurs inside the transmembrane domain and is catalyzed by the γ-secretase complex, which releases NICD for translocation to the nucleus. In the nucleus, NICD binds to the DNA-binding protein RBP-J, leading to Notch target genes’ transcription. CSL is a DNA-binding protein that recruits NICD to Notch target gene promoters. Schematic presentation of the PI3K/Akt/mTOR signaling pathway: Ligands bind to the receptor tyrosine kinases like Her2, which leads to conformational changes and activates PI3K. PI3K initiates activation of Akt by phosphorylation, which acts as a major activation source to further downstream signaling moieties involved in various cellular processes (mTOR).

HER2 belongs to the HER family of receptor tyrosine kinase, and the most common effectors include the MAPK pathway, PI3K/Akt signaling pathway, and PKC activation (Figure 1). These signaling pathways involve cell proliferation, differentiation, survival, adhesion, migration, and apoptosis [136]. HER2 gene amplification is reported in many breast cancer patients and is associated with poor clinical prognosis [137].

A number of studies have highlighted the central role of altered HER2 signaling in BCSC maintenance/enrichment and explain its bidirectional communication with Notch and Wingless/β-catenin pathways. d16HER2 is a splicing variant of HER2 that has been identified as one of the most oncogenic HER2 isoforms, mediating EMT/stemness and response to targeted therapy. Moreover, the expression of p95HER2, a HER2 fragment that can regulate CSC characteristics, was identified in HER2-positive breast cancers with poor prognosis [138].

The treatment strategy based on HER2 inhibitors, trastuzumab, pertuzumab, lapatinib, and TDM-1, is used in HER2-positive BC [139,140,141,142,143,144,145,146,147]. Trastuzumab, a humanized IgG1 monoclonal antibody, targets the extracellular domain of the HER2 and is a standard, first-line treatment for metastatic and early-stage, HR+/HR−, HER2-positive breast cancer in combination with chemotherapy [138]. In addition, in conjunction with pertuzumab, deruxtecan, or T-DM1 is recommended as a second-line strategy [148]. Pertuzumab is a recombinant, humanized, monoclonal antibody that binds to the HER2 extracellular dimerization domain II. It inhibits heterodimerization of HER2 with HER1, HER3, HER4, and IGF-1R31 and, in consequence, decreases tumor cell growth. The combination of trastuzumab and pertuzumab can be used for the treatment of HER2-positive, metastatic breast cancer [141]. Lapatinib is a tyrosine kinase inhibitor that blocks the phosphorylation of HER1 and HER2. The combination therapy of lapatinib and capecitabine increased the survival rate of HER2-positive MBC patients who were previously treated with trastuzumab and did not respond to therapy [149]. Trastuzumab emtansine (T-DM1) is an antibody conjugate of drugs: trastuzumab and emtansine (DM1), a microtubule inhibitor. TDM1 possesses trastuzumab activity and ensures intracellular DM1 delivery to HER2 overexpressing cells. This drug is used in patients with HER2-positive metastatic breast cancer who previously received trastuzumab and a taxane [147] (Table 3).

Table 3 presents different drugs used in BCSC elimination, divided by signaling pathways targeting BCSCs and different molecular subtypes of breast cancer.

**Table 3:** Treatment strategy for eliminating BCSCs, including signaling pathways targeting BCSCs and individual molecular subtypes of breast cancer [71,72,73,74,75,76,77,78,85,96,99,102,103,104,105,106,107,108,109,110,111,115,116,117,118,119,120,121,122,123,124,124,125,126,127,128,129,130,131,132,133,133,134,138,138,139,140,141,141,142,143,144,145,146,147,148,149].

	Signaling Pathways Targeting BCSCs				
BC Subtype	Wnt	Notch	Hedgehog	PI3K/AKT/mTOR	HER2
Luminal		MK-0752	Vismodegib	Alpelisib	
		RO-4929097	GANT61	Everolimus	
				XL147	
				NVP-BKM120	
				LY-294002	
				Perifosine	
				Flubendazole	
HER2+					Trastuzumab
					Pertuzumab
					Lapatinib
					TDM-1
TNBC	LGK-974	MK-0752	Vismodegib		
		PF-03084014	Sonidegib		
		RO-4929097	GANT61		
		nirogacestat			
Basal/ ER- HER2−	Foxy-5		Sonidegib	GDC-0941	
	Cirmtuzumab				
	Vantictumab				

#### 4.1.6. Active Clinical Trials

One of the currently active clinical trials based on an extremely interesting concept involves EpCAM CAR-T, used for the treatment of advanced solid tumors, including breast cancers. It verifies the safety of T cells with chimeric antigen receptor (CAR-T)-identifying EpCAM. EpCAM is a well-known marker associated with metastatic and invasive tumors and is correlated with poor prognosis (NCT02915445). Additionally, a clinical trial with the Wnt signaling pathway ligand LGK974, in combination with anti-PD-1 spartalizumab (PDR001), was targeted for TNBC patients who are either naive or primary refractory to prior anti-PD-1 therapy (NCT01351103). Spartalizumab is a monoclonal antibody and checkpoint inhibitor, directed against the negative immunoregulatory human cell surface receptor, programmed death-1 (PD-1). Another already completed pilot phase 1b study used cirmtuzumab in combination with paclitaxel and indicated that the combination of these drugs is safe and well tolerated in patients with metastatic HER2-negative or locally advanced, unresectable BC (NCT027776917). A combination of paclitaxel with reparixin, an inhibitor of CXCR1, which is the target of BCSCs, was applied in patients with metastatic TNBC. This study revealed that the treatment is safe and tolerable for patients (Frida trial, NCT02370238).

## 5. Conclusions

The enormous diversity of breast cancers is a great challenge, but on the other hand, it can be used to find new treatment options. While the vast amount of data can be confusing, certainly, the more we know, the better for the patient. Awareness of a specific panel of molecular features, biomarkers, and genetic variations in different cancer subtypes gives a much better understanding of their biology. This can prompt further research, such as new signal transduction pathways in breast cancer development. In addition, this knowledge has significant prognostic value for patients. Ultimately, it indicates an appropriate and effective treatment strategy, especially when we know about the presence of different subpopulations, including cancer cells and cancer stem cells in the tumor. Therefore, targeting both cell types is the ideal strategy for eliminating this disease. This holistic approach is essential and extremely important for improving therapeutic outcomes, preventing relapse, and eliminating cancer as a whole.

## Figures and Tables

**Table 1:** Characteristics of individual breast cancer subtypes, including frequency, immunohistochemistry characteristics, and prognosis, according to [33].

Molecular Subtype	Frequency	IHC Characteristics	Prognosis
Luminal A	40%	ER+/PR+/Ki-67-low	Very good
Luminal B	20%	ER+/PR+/Ki-67-high	Good
HER2-enriched	10–15%	ER−/PR−/HER2+	Poor
Basal/TNBC	15–20%	ER−/PR−/HER2−	Very poor

**Table 2:** Characteristics of BCSC biomarker patterns in different molecular subtypes of breast cancer. ALDH1—aldehyde dehydrogenase 1; TNBC—triple negative breast cancer; BL1—basal-like 1; BL-2—basal like 2; M—mesenchymal. √—presence of biomarker, - lack of biomarker.

	BCSC Subtypes				
Biomarkers	Luminal A	Luminal B	HER-2	Basal	TNBC
CD44+/CD24−	-	-	-	√	√ BL2, M
CD133	√	√	√	√	√
ALDH1+	-	√	-	√	√ BL1
miR-200a	√	-	-	-	-
miR-200c	√	-	-	-	-
miR-9	-	-	√	-	√
miR-141	√	-	-	-	-
miR-155	-	-	-	-	√

## Abbreviations

ABCG2	ATP-binding cassette subfamily G member 2
ADAM	a disintegrin and metalloproteinase protein
Ais	aromatase inhibitors
AKT	a serine/threonine protein kinase
ALDH1	aldehyde dehydrogenase 1
APC	adenomatous polyposis coli
BC	breast cancer
BCSCs	breast cancer stem cells
BL1	basal-like 1 subtype of TNBC
BL2	basal-like 2 subtype of TNBC
BLBC	basal-like breast cancer
BLIA	basal-like immune-activated subtype of TNBC
BLIS	basal-like immune-suppressed subtype of TNBC
BRCA1	BReast CAncer gene 1
BRCA2	BReast CAncer gene 2
CB-103/LIMANTRAFIN	an orally active inhibitor of the Notch transcription activation complex
CD24	a cluster of differentiation 24
CD44	a cluster of differentiation 44
CD70	a cluster of differentiation 70
CD133	a cluster of differentiation 133
CK	cytokeratin
CK1	casein kinase 1
CSC	cancer stem cells
CSL	CBF1, Suppressor of Hairless, Lag-1
CTFs	carboxy-terminal truncations
Dsh	Disheveled
EGFR	epidermal growth factor receptor
EMT	epithelial–mesenchymal transition
EPCAM	epithelial cell adhesion molecule
ER	estrogen receptor
ESMO	European Society for Medical Oncology
FUSCC	Fudan University Shanghai Cancer Center
FZD	Frizzled proteins
GANT61	an inhibitor for GLI1 as well as GLI2-induced transcription
GDC-0941 (Pictilisib)	a potent inhibitor of PI3Kα/δ
GLI	glioma-associated oncogene
GLI2/3R	GLI2/3 repressors
GLIACT	GLI activators
GSK3β	glycogen synthase kinase 3β
HER2	human epidermal growth factor 2
Hh	Hedgehog
IM	an immunomodulatory subtype of TNBC
LAR	luminal androgen receptor subtype of TNBC
LEF	lymphatic enhancer factor
LGK-974	specific PORCN inhibitor
lncRNA	long noncoding
Lrg5	leucine-rich repeat-containing G protein-coupled receptor 5
LY-294002	a PI3K inhibitor
M	mesenchymal subtype of TNBC
MaSCs	mammary gland stem cells
MBC	metastatic breast cancer
MES	mesenchymal subtype of TNBC
MET	mesenchymal–epithelial transition
MK-0752	γ-secretase inhibitor
MS	mammosphere
MSL	mesenchymal stem-like subtype of TNBC
mTOR	mammalian target of rapamycin
MUC1	mucin 1
NICD	Notch intracellular domain
NVP-BKM120 (Buparlisib)	a pan-class I PI3K inhibitor
PD-L1	programmed death-ligand 1
PF-03084014	γ secretase inhibitor
PI3K	phosphatidylinositol 3-kinase
PIK3CA	phosphatidylinositol-4,5-bisphosphate 3-kinase catalytic subunit alpha
PKA	protein kinase A
PR	progesterone receptor
PTCH1	Patched 1
RBP-J	recombining binding protein J
RO-4929097 (RG-4733)	γ secretase inhibitor
SMO	Smoothened SOX9 SRY-box transcription factor 9
SSEA-3	stage-specific embryonic antigen-3
STAT	signal transducer and activator of transcription
SUFU	suppressor of fused homolog
TCF	T-cell factor
T-DM1	Trastuzumab emtansine
TICs	tumor-initiating cells
TNBC	triple-negative breast cancer
TWIST	Twist-related protein 1
WNT5A	Wnt family member 5A
XL147 (Pilaralisib)	a potent and highly selective class I PI3K inhibitor
